# Roller Bearing Performance Degradation Assessment Based on Fusion of Multiple Features of Electrostatic Sensors

**DOI:** 10.3390/s19040824

**Published:** 2019-02-17

**Authors:** Ying Zhang, Anchen Wang, Hongfu Zuo

**Affiliations:** 1 College of Automobile and Traffic Engineering, Nanjing Forestry University, Nanjing 210037, China; wac646708214@163.com; 2 College of Civil Aviation, Nanjing University of Aeronautics and Astronautics, Nanjing 210037, China; rms@nuaa.edu.cn

**Keywords:** roller bearing, electrostatic monitoring, spectral regression, gaussian mixture model, Bayesian inference distance

## Abstract

This paper presents a new method to assess the performance degradation of roller bearings based on the fusion of multiple features, with the aim of improving the early degradation detection ability of the electrostatic monitoring system. At first, a set of feature parameters of the electrostatic monitoring system indicating the normal state of the bearings are extracted from the perspective of the time domain, frequency domain and complexity. Then, the parameter set is processed to reduce the dimensions and eliminate the redundancy using spectral regression. With the processed features, a Gaussian mixed model is established to gauge the health of the bearing, providing the distance value obtained using Bayesian inference as a quantitative indicator for assessing the performance degradation. The method is applied to access the life of a bearing in which the mechanic fatigue is artificially accelerated. The test results show that the proposed method can better reflect the degradation process of the bearing compared to other evaluation methods. This enables the electrostatic monitoring technique to detect the degradation of the bearing earlier than the vibration monitoring, providing a powerful tool for the condition monitoring of roller bearings.

## 1. Introduction

Rolling bearings are one of the key components in rotating machinery, the degradation or failure of which will affect the performance of the whole machine, or even cause unplanned outage, resulting in economic losses or even mass casualties; therefore, its condition monitoring and diagnosis have always been a research focus [1]. Henao summarized the application of the current signature analysis and advanced digital signal processing method in the diagnosis of electrical machines [2]. Frosini proposed a novel technique based on the stray flux measurement in different positions around the electrical machine [3]. Immovilli compared the bearing fault detection capability obtained with the vibration and current signals [4].

At present, a high-sensitivity monitoring technique based on electrostatic induction has emerged as a promising technique for the condition monitoring of roller bearings. It is based on electrostatic induction, and the advantage of this technique is that it measures a direct product of the fault, rather than secondary effects, such as increased vibration or temperature exceedance [5]. Thus, it has the ability to detect faults and component deterioration earlier than the traditional monitoring techniques. Harvey and Craig [5,6] implemented electrostatic wear-site sensors on a bearing test rig to evaluate their effectiveness in detecting bearing faults, using the root mean square (RMS) value of the electrostatic sensors to evaluate the degenerate state of the bearings, and the results showed that the electrostatic sensors could detect abnormalities earlier than vibration. In our previous work, an electrostatic sensor was applied to the online monitoring experiments of combustor carbon deposition faults in aero-engines, and the results showed that the baseline model-based fault-detection method was capable of detecting the gas-path component fault in an on-line fashion [7,8]. We designed a wear-site electrostatic sensor and verified its performance by testing roller bearings; the results showed that electrostatic sensors could effectively detect the early failure of bearings [9,10].

The electrostatic monitoring technique has been proved to be an effective tool in the condition monitoring of roller bearings. However, further improvements of its early detection ability are necessary in the following two areas: (1) the existing methods of extracting features from the electrostatic signals are conventional in the time domain and frequency domain (such as the RMS value, frequency of fault feature, etc.), which cannot completely reflect the operational status of the bearings with high sensitivity, especially regarding early degradation; (2) the method of building a fusing quantitative indicator of the roller bearing performance degradation, which will help maintenance staff to determine the operational status of the bearing and make proper maintenance decisions accordingly. In terms of the first problem, some feature extraction methods have been developed, especially in the frequency domain of acoustic emission monitoring and vibration monitoring, which is very efficient in incipient bearing fault detection. Farzad Hemmati [11] proposed a new approach of optimizing wavelet functions based on maximizing the Kurtosis-to-Shannon entropy ratio (KER) value, and selecting the optimal band pass filter signals to detect incipient faults. Farzad Hemmati [12] proposed a new method based on optimizing the ratio of Kurtosis, Shannon entropy and the wavelet packet transform to diagnose localized defects on rolling element bearings. As the proportion of random components in the electrostatic monitoring signal changes continuously throughout the life cycle of the roller bearing, it is feasible to introduce a complexity measurement method to describe this change, which will help improve the early fault detection ability [13]. In terms of the second problem, it is feasible to make use of the bearing performance evaluation methods based on the vibration monitoring as they have become very mature and effective as a result of in-depth research. There are two key issues in the bearing performance assessment. One of the issues is to accurately extract useful information reflecting the operational status of the device from the high-dimensional and non-linear data to avoid data redundancy and conflicts. The principal component analysis (PCA) method is a widely used feature extraction method which realizes the classification by extracting the global maximum variance information [14]. The manifold learning algorithm recovers the low-dimensional manifold structure from the high-dimensional sampling data, resulting in a reduction in the data dimension. A local preservation projection (LPP) method was proposed that preserves the local manifold structure of the data set and obtains the mapping results of test data, which is suitable for online learning [15]. However, this method requires the feature decomposition of dense matrices. If the matrix dimension is high, it will influence the time taken for learning and dimension reduction. To address this problem, Cai proposed a spectral regression (SR) algorithm to solve the projection function using a regression framework, thus avoiding the feature decomposition problem of dense matrices [16]. Another question is how to conduct a quantitative assessment of the performance degradation of bearings based on the extracted feature parameters. Yu applied the Gaussian mixture model (GMM) model to the health assessment of bearings and achieved the quantitative analysis of the performance degradation of the bearings [17]. Zhang proposed an exergy-based fusion method to fuse multiple transient states with the information of multiple sensors obtained in state monitoring [18]. Shao proposed a feature fusion method based on the deep-belief network (DBN), taking advantage of the excellent learning ability of DBN [19]. Jiang and Chen presented an intelligent bearing performance degradation assessment method based on hidden Markov model (HMM) and the nuisance attribute projection (NAP) to obtain good performance evaluation results [20]. Akhand Rai proposed a novel method for bearing performance degradation assessment based on an amalgamation of empirical mode decomposition (EMD) and k-medoids clustering; the results demonstrated that the method could confirm the early stage defect [21]. Wang constructed a generalized dimensionless bearing health indicator based on the principle of squared envelope analysis; the results showed that the generalized dimensionless health indicator is sensitive to an incipient bearing defect [22]. All of the above methods have yielded good assessment results.

To address the issues mentioned above, a new performance degradation assessment algorithm for the electrostatic monitoring of roller bearings is put forward. At first, a set of feature parameters are extracted in the time domain, frequency domain and the complexity of the signals. Then, the obtained features will undergo the dimensional reduction process using spectral regression. A Gaussian mixed model (GMM) model is established to gauge the health of the bearing, with an indicator obtained based on Bayesian inference distance serving as a quantitative measure of the bearing performance degradation. The effectiveness of the proposed method is verified by a bearing accelerated fatigue life test.

## 2. Feature Extraction

### 2.1. Traditional Features

The traditional features of the electrostatic signals are listed in Table 1, which could be found in the vibration measures and our previous work [9]. The bearing defect frequencies of the electrostatic signals are given by Chen [23] and our previous work [9], which are similar to the vibration measurement.

### 2.2. Permutation Entropy (PE)

Permutation entropy (PE) is a complexity measure for time series based on comparing neighboring values and was introduced by Bandt and Pompe [24]. The first step is the phase-space reconstruction in the time series X=[x(1),x(2),x(3),…,x(N)]:

(1) $$Y=\left[\begin{array}{cccc} x(1) & x(1+\tau ) & \ldots & x(1+(m-1)\tau )\\ \ldots & \ldots & \ldots & \ldots \\ x(i) & x(i+\tau ) & \ldots & x(i+(m-1)\tau )\\ \ldots & \ldots & \ldots & \ldots \\ x(N-(m-1)\tau ) & x(N-(m-2)\tau ) & \ldots & x(N) \end{array}\right]$$

where *m* is the embedded dimension and τ is the time delay.

Rearrange each row of the reconstructed matrix *Y* by the ascending sequence as

(2) $$\left\{x(i+(j_{1}-1)\tau )\leq x(i+(j_{2}-1)\tau )\leq \ldots \leq x(i+(j_{m}-1)\tau ))\right\}$$

where $j_{1},j_{2},\ldots ,j_{m}$ are the row indexes of each element in the reconstructed components. If there are equal values in the reconstructed components, e.g., $x(i+(j_{p}-1)\tau )=x(i+(j_{q}-1)\tau )$, the series is then sorted by the sizes of $j_{p}$ and $j_{q}$ values, which means that, when $j_{p}\leq j_{q}$, $x(i+(j_{p}-1)\tau )\leq x(i+(j_{q}-1)\tau )$. Therefore, any matrix *Y* can be mapped onto a group of symbols as $S(l)=(j_{1},j_{2},\ldots ,j_{m})$, where l=1,2,…k and k≤m!. *M* different symbols have m! different arrangements in total; i.e., m! different symbol sequences. Calculate the probability of the occurrence of each symbol sequence $P_{1},P_{2},\ldots ,P_{k}$, and then the permutation entropy of the *k* different symbol sequences of the time series *X* can be defined in the form of Shannon entropy: $H_{P}=H_{P}(m)/(m-1)$. PE has a practically important invariance property. When Gaussian noise was added to the deterministic time series, the observational noise causes only a small increase of PE [24,25], and the advantage of permutation entropy is that it can be applied to the real-time on-line monitoring of bearings because of its simple algorithm and fast computation. $H_{P}$ describes the local order structure of the time series; the smaller the $H_{P}$ value, the more regular the time sequence is. Conversely, the greater the $H_{P}$ value, the more random the time series is. Given that the impacts of the nonlinear factors of a system device are different when abnormalities or faults are about to occur, the signal complexity will be different as well. Therefore, it is feasible to characterize the occurrence and development of device faults with permutation entropy. The permutation entropy algorithm has three parameters to be defined: data length w, embedding dimension m and delay time τ. The selection rule of the three parameters has been analyzed in detail in [26]. In our previous work [9], we found that PE could significantly bring forward the time when early faults are detected in electrostatic monitoring sensors, and PE can be used as a useful supplement to the features of electrostatic monitoring.

### 2.3. Spectrum Regression, SR

For a given data set $z_{1},z_{2},\ldots ,z_{m}\in R^{n}$, the purpose of spectral regression is to find the transformation matrix *A* so that the mapping $h_{1},h_{2},\ldots ,h_{m}\in R^{l}\left(l<<n\right)$ can be found in the low-dimensional space for a given point *m*, which is $h_{i}=A^{T}z_{i}$.

A graph *G* consisting of *m* vertices is established from the perspective of graph embedding, where the *i*th point is $z_{i}$. Define *W* as a symmetrical weight matrix, in which element $W_{ij}$ is the weight connecting the two points *i* and *j*. Spectral regression seeks a linear projection function through two steps: (i) solving the equation Wy=λDy, where the feature vectors $y_{0},y_{1},\ldots ,y_{l-1}$ corresponding to the first *l* features are selected, and *D* is the diagonal matrix $D_{ii}=\sum_{j}W_{ji}$; (ii) obtaining the projection vector *a* by solving the following regular least squares:

(3) $$a=\underset{a}{\arg\min}\left(\sum_{i=1}^{m}{\left(a^{T}z_{i}-y_{i}\right)}^{2}+\lambda {\left\|a\right\|}^{2}\right)$$

where λ is a regularization parameter. Therefore, the original data can be reduced to an *l*-dimension vector:

(4) $$z\to h=A^{T}z,\;A=\left(a_{0},a_{1},\ldots ,a_{l-1}\right)$$

## 3. Performance Degradation Assessment Model with the Fusion of Multiple Features Based on GMM

### 3.1. Gaussian Mixture Model (GMM)

The Gaussian mixture model approximates the arbitrary distribution through several weighted combinations of single Gaussian components:

(5) $$p(x)=\sum_{m=1}^{M}\pi_{m}p(x|\theta_{m})$$

where $x={\left[x_{1},\ldots ,x_{l}\right]}^{T}$ is the *l*-dimensional data vector, *M* is the number of mixture components, $\pi_{m}$ are the mixing proportions subject to $\sum \pi_{m}=1$, each component density $p(x|\theta_{m})$ is a normal probability distribution, the mean vector is $\mu_{m}$ and the covariance matrix is $S_{m}$. These parameters are encapsulated into a parameter vector to get $\phi =\{\pi_{1},\ldots ,\pi_{m};\mu_{1},\ldots ,\mu_{m};S_{1},\ldots S_{m}\}$. The usual choice for obtaining the optimum vector of the mixture parameters is the expectation–maximization (EM) algorithm [27].

After the baseline GMM model is established based on the normal state data, a quantitative indicator based on the Bayesian inference is employed to conduct the quantitative indicator of the bearing performance degradation assessment.

Assume that there are *K* Gaussian components. The *k*-th component is denoted as $C_{k}$, and its probability of occurrence is $\alpha_{k}$. Then, for the test point $x_{t}$, the probability of the *k*-th component $C_{k}$ is $p(C_{k}|x_{t})$, which can be calculated using Bayesian inference as follows:

(6) $$p(C_{k}|x_{t})=\alpha_{k}p(x_{t}|C_{k})/p(x_{t})=\alpha_{k}p(x_{t}|C_{k})/\sum_{i=1}^{K}\alpha_{i}p(x_{t}|C_{k})$$

where the value of $\alpha_{k}$ can be obtained from the modeling data, which is a prior probability. $p(x_{t}|C_{k})$ can be calculated by the following formula:

(7) $$p(x_{t}|C_{k})=\frac{1}{{\left(2\pi \right)}^{1/2}|{S_{k}|}^{1/2}}\exp\left[-\frac{1}{2}{\left(x_{t}-\mu_{k}\right)}^{T}S_{k}^{-1}\left(x_{t}-\mu_{k}\right)\right]$$

where $\mu_{k}$ and $S_{k}$ represent the mean and covariance matrices of the *k*-th Gaussian component, respectively. Further, defining the distance of $x_{t}$ to each component $C_{k}$:

(8) $$D_{local}^{C_{k}}(x_{t})={\left(x_{t}-\mu_{k}\right)}^{T}S_{k}^{-1}(x_{t}-\mu_{k})$$

The global indicator of the input data, called the Bayesian inference distance (BID) indicator, can be obtained by calculating the weighted sum of distances of all components.

(9) $$BID=\sum_{k=1}^{K}p(C_{k}|x_{t})D_{local}^{C_{k}}(x_{t})$$

### 3.2. Performance Assessment Model

**Figure 1:**
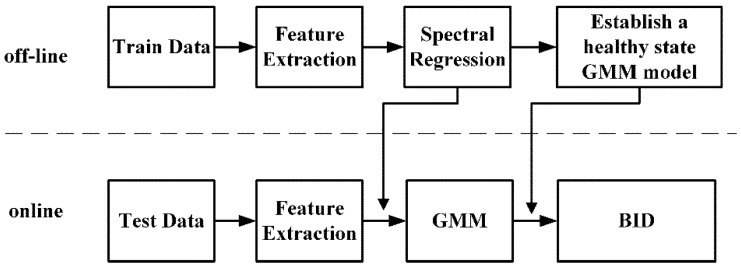
Flowchart of proposed performance degradation assessment method. GMM: Gaussian mixture model; BID: Bayesian inference distance.

Based on the above analysis, a new performance degradation assessment algorithm is proposed for the electrostatic monitoring of roller bearings. The process is shown in Figure 1, which consists of two parts: off-line modeling and on-line evaluation.

**Off-line:**

(1) Extract the features and use the SR method to reduce the dimensions of the original feature space;
(2) Select the normal state data to establish the GMM model and determine the model parameters;
(3) Calculate the BID. The sliding average method is used to smooth the indicator and improve the sensitivity and reliability of the indicator:
  (10) $$y(i)=1/M\sum_{j=0}^{M-1}x(i+j)$$
  where x(i) is the input signal; y(i) the output signal; and *M* is the number of average sliding points, which is considered as 5 in this paper. We implemented a prior experiment that used more than 5 sliding points, and the results show that it does not detect abnormities as early as possible. We also used less than 5 sliding points, and the results showed that it cannot reflect the trend of degradation as well as the 5-point sliding average;
(4) Establish the control line. To be able to set off an alarm when a slight degradation occurs, a control line needs to be established based on the kernel density estimation (KDE) [28]. When the performance degradation occurs, an alarm will be triggered. There are some kernel functions used for KDE; in this paper, the Gaussian kernel function is often used. Depending on the confidence level required, 99% (i.e., the false alarm rate is 1% for healthy bearing), the threshold BID can be calculated to define the confidence bound.

**Online:**

(1) Based on step 1 of off-line modeling, the features are extracted and dimensional reduction is performed;
(2) Calculate the BID distance between the test data and the normal state GMM model;
(3) Perform a quantitative assessment of the bearing performance and determine the condition of bearings.

## 4. Experimental Results

In this section, a bearing accelerated life test is carried out to investigate the effectiveness of the proposed approach in this paper.

### 4.1. Test Rig

**Figure 2:**
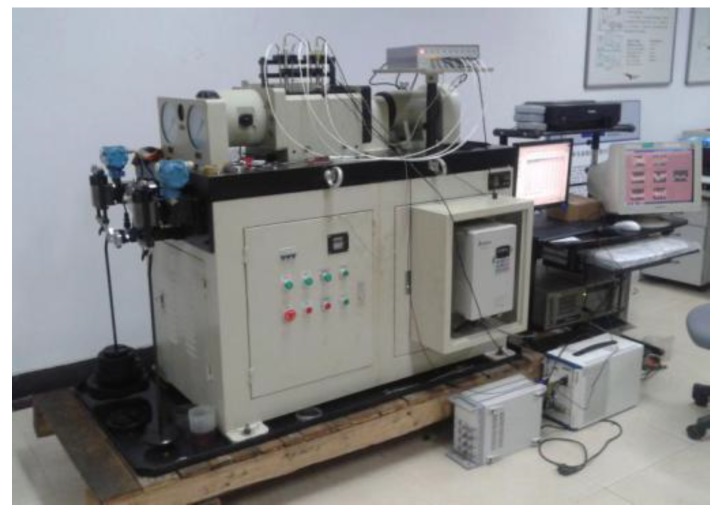
Test rig.

**Figure 3:**
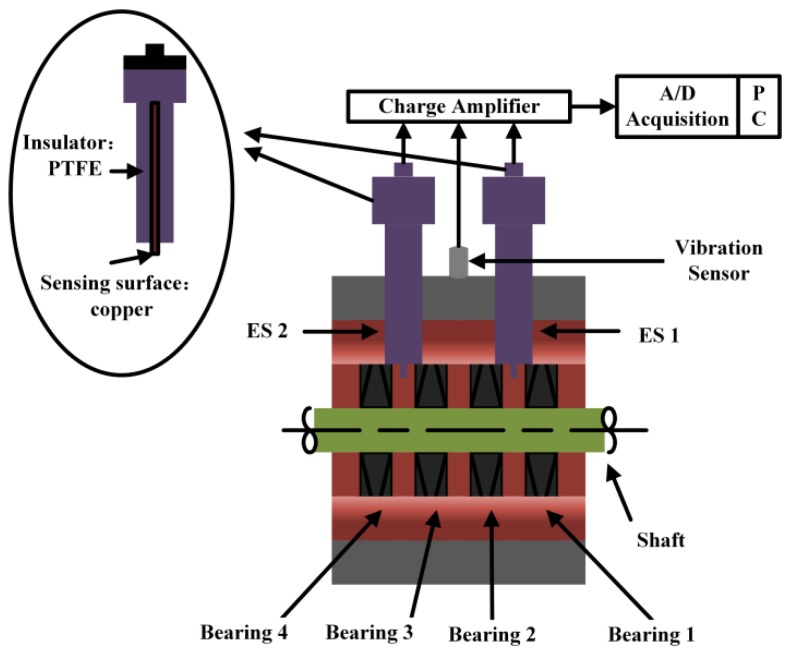
Schematic of the structure of the electrostatic sensor, the mounting position of monitoring sensors and the acquisition systems.

The full life cycle test of the rolling bearing is conducted on the ABLT-1A provided by Hangzhou Bearing Test and Research Center (HBRC). The test rig is shown in Figure 2. The test chamber accommodates four bearings driven by a DC motor; the test will stop when the accumulated debris adhered to the magnetic plug exceeds a certain level and causes a switch to turn off. These electrostatic sensors consist of a sensing surface copper, which is surrounded by an insulator polytetrafluoroethylene (PTFE); the outer body acts as a shield to minimize noise. The structure of the electrostatic sensors, installation position and data acquisition system are shown in Figure 3; details of the electrostatic sensors and the experiment can be seen in our previous work [9].

**Figure 4:**
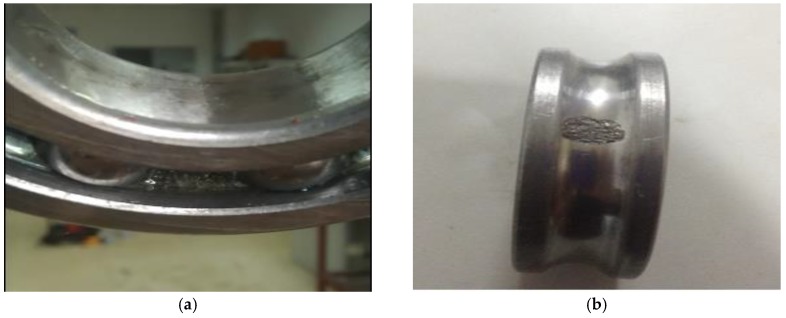
The defective surface of failure bearings: (**a**) outer-race fault, (**b**) inner-race fault.

Two electrostatic sensors (ES1 and ES2) are used for monitoring (ES1 for bearings 1 and 2, ES2 for bearings 3 and 4). When charged particles pass through the effective area in front of the electrostatic sensor, the electric field line will terminate at the probe surface. Due to the effect of electrostatic induction, the opposite polar charge is attracted on the probe surface. Then, the same polar charge is driven to the other end of the probe surface. Since the other end of the probe is connected with the signal conditioning circuit, a measurable output voltage signal is formed. When the bearing fails, large amounts of debris will be produced and hugely modified contact surfaces generate a high charge, which will be reflected by the electrostatic sensor. Other conditions are set as follows: test bearing type, 6207, the roller diameter of the test bearing is 11.1125 mm, the bearing raceway (cage) diameter is 53.5 mm, the contact angle is 0, the rolling number is 9, the experimental radial load is 20 kN, the rotation speed is 3000 r/min, the sampling frequency is 10 kHz, the data length is 4096 points, and one group’s data are collected every 1 min. Four tests (i.e., test 1–4, where each test has four bearings) were implemented in this experiment. We used bearing 2 of test 1 (B1) and bearing 3 of test 2 (B2), as our experimental bearings, with wear-out failures found on the outer race of B1 (Figure 4a) and inner race of B2 (Figure 4b).

### 4.2. Classification of Degradation Degrees

**Figure 5:**
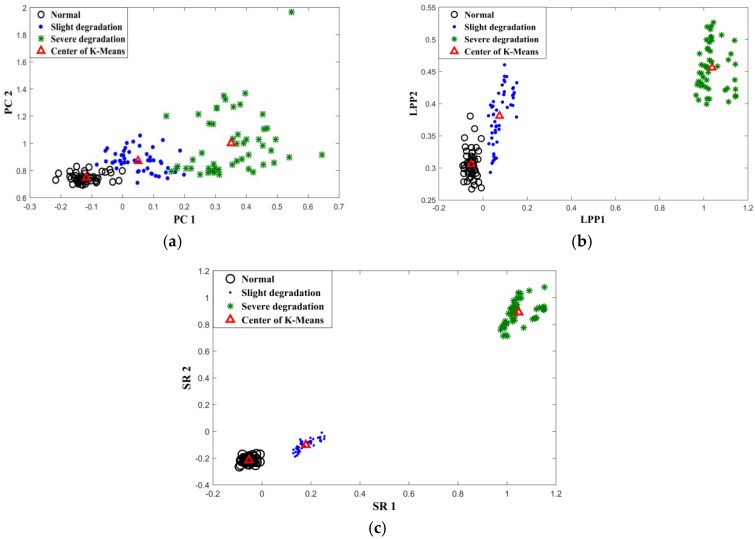
Projected data point distribution with vectors [y1, y2], (**a**) PCA, (**b**) LPP, (**c**) spectral regression (SR).

The objective of the performance degradation assessment is to detect the occurrence of degradation as early as possible. This requires extracting useful information from the multidimensional feature set that reflects specific degradation with high sensitivity. In this study, data sets of normal state, slight degradation date and severe degradation date are extracted (100 sets each). Among this, 50% of the data is used for training and 50% for testing. The aim is to verify the classification and generalization capabilities of the SR feature extraction technique proposed in this section. The 15 original features which are composed of the traditional features and PE values are extracted from the training data, and then the SR method is performed on the vectors. The first eigenvector, corresponding to the largest eigenvalue, is selected for low-dimensional mapping. Generally, the first few eigenvectors (corresponding to the maximum eigenvalues) will maintain the maximum variance information in the given data; the selection of the number of the eigenvectors should be based on the actual application requirements. In this paper, we finally chose the first 2 eigenvectors referring to the literature [17,28], which will not increase the computational complexity, but also can result in a better visual display. For comparison, PCA and LPP are employed separately for feature extraction. Figure 5 is the diagram plotted when the first two dimensions were used. It can be seen from this figure that the SR method is better compared to LPP and PCA at distinguishing different degrees of degradation. The dots of each type are more concentrated in the SR method. Although the LPP method can distinguish between different states, the dots are relatively scattered. In particular, there is no clear-cut distinction between the normal state and slightly degraded state. To illustrate the effectiveness of the three feature extraction methods, the K-mean is used as the classifier for degradation. The testing results are shown in Table 2, in which the accuracy rate calculates the proportion of correct classifications and the computation time is measured under the computer environment (MATLAB, Intel i5 CPU, 8 G RAM). It can be seen from the table that the use of SR for feature extraction achieved the highest detection rate. Also, SR is superior in terms of computing time. This experiment demonstrates that the feature extraction using SR method is more efficient in the classification of degradation degree and faster in computing. Therefore, it is more suitable for on-line monitoring.

### 4.3. Bearing Performance Degradation Assessment

**Figure 6:**
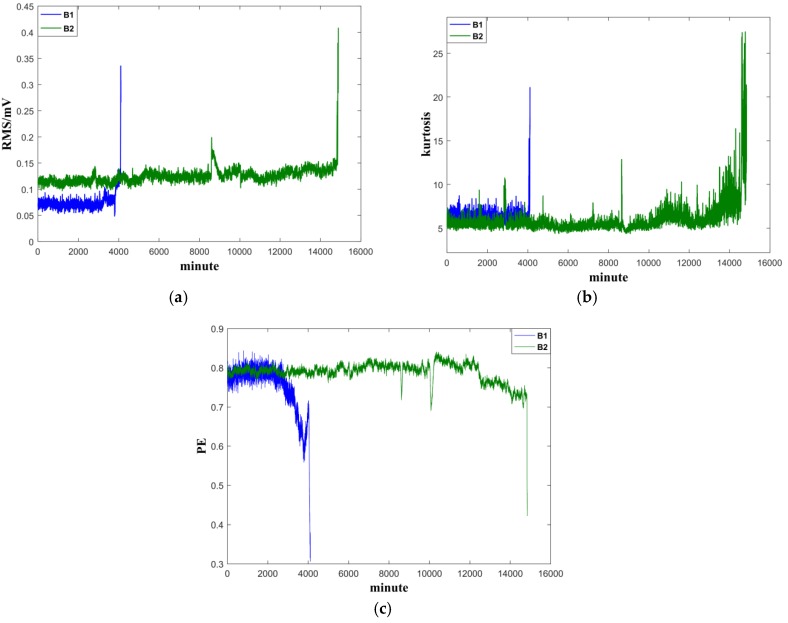
Features of the tested bearings on their full cycle life: (**a**) RMS, (**b**) kurtosis, (**c**) PE.

Three representative features—RMS, kurtosis and permutation entropy—are chosen for in-depth demonstration. According to the results in the literature [26], the data length is set to 128, and the number of reconstructed dimensions and the delay time are set to m=6, τ=3, respectively. The PE values of a file (1 min) will be the average of the 32 parts. The variations of the three values are shown in Figure 6. The following observations can be made from the figure: (1) the PE values obtained from the two experiments are consistent. The PE of B1 indicates early degradation earlier than RMS and kurtosis, and the PE of B2 shows more salient trends of degradation; (2) the kurtosis of B2 fluctuates in a wide range at the early stage. The PE of B1 and the RMS of B2 show a slight upward trend in the early stage. These phenomena make it difficult to make an accurate judgment of bearing degradation and could trigger a false alarm, leading to incorrect maintenance decisions; (3) different indicators indicate faults (change trends) at different stages. Indicators such as PE are more sensitive to early degeneration; therefore, it is important to have an effective quantitative index that can integrate all features information, so that it can be used to assess the degradation of bearing performance continuously and reliably.

**Figure 7:**
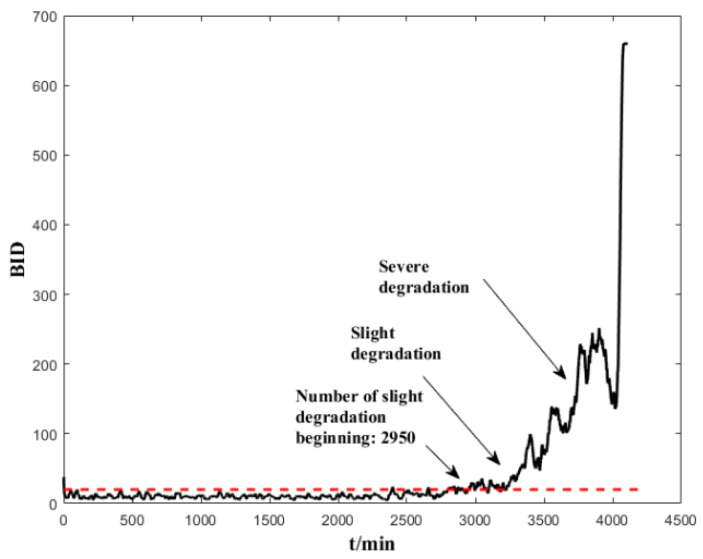
Performance assessment (BID) on the whole life time of B1.

**Figure 8:**
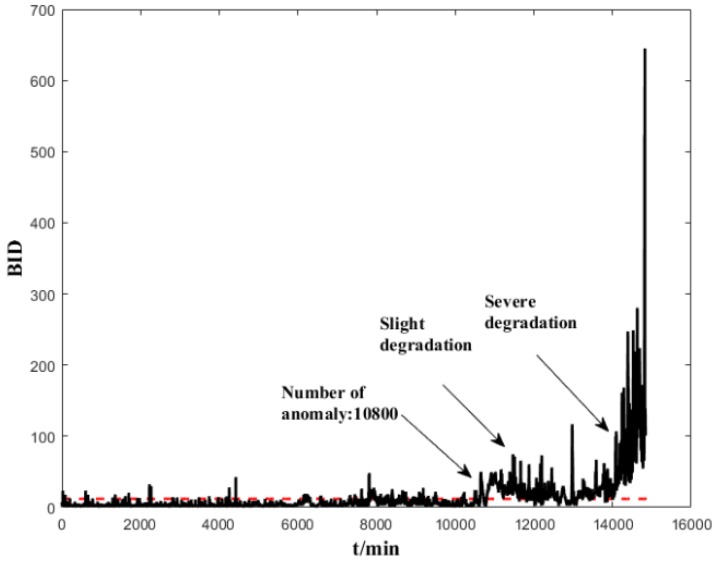
Performance assessment (BID) on the whole life time of B2.

The normal-state data (200 units) are used to establish a healthy status GMM model. Several trials show that a GMM with 5 Gaussian components is sufficient to model the normal-state data. We implemented a prior experiment that used more than 5 Gaussian components (6,7,8) and the results show that it cannot improve the early failure detection capability of ES sensors, but increases the computing time. We also used less than 5 Gaussian components (2,3,4), and the results show that the degradation trend does not perform as well as with 5 Gaussian components. After the dimensional reduction through spectral regression, the first three eigenvectors corresponding to the largest three eigenvalues are selected as input data. The parameters of the GMM are initialized using K-means, and the maximum number of iterations is set as 1000. After the health-status GMM models are established, each subsequent input data will have a corresponding BID value. The corresponding results are shown in Figure 7 and Figure 8. It can be seen from these figures that the indicators are stable and can clearly reflect the changes in the life cycle of the bearing. The BID value fluctuated slightly in the normal state. B1 began to enter the early stages of degeneration after 2950 min, deteriorated sharply and entered the stage of serious degradation after 3700 min before complete failure. B2 began to enter the early degeneration stage after 10,800 min, deteriorated sharply and entered the stage of serious degradation after 14,000 min before complete failure.

The BID value of the electrostatic signal has been presented obviously with regard to health, light degradation, and severe degradation to failure. As BID fuses the characteristic parameters, BID reflects the charge level in the wear area of the bearing comprehensively. The level of the charge indirectly reflects the wear state of the bearing; that is, when severe wear occurs to the bearing, the charge in the wear area increases, the BID rises sharply, and a drastic change can be seen from the vibration signal. When the BID value increases slowly, the level of charge increases slowly, and a certain degree of degradation occurs; the vibration level does not change significantly in this period. From the experimental data, there is a short period from the severe degradation occurrence of an incipient defect to final failure; this is also verified in the literature [5,29]. The advantage of electrostatic monitoring is to detect anomalies as early as possible before the bearings fail completely, thus providing sufficient time for maintenance personnel to make maintenance decisions.

## 5. Comparison and Analysis

### 5.1. Comparison with the Other Two GMM-Based Indicators

**Figure 9:**
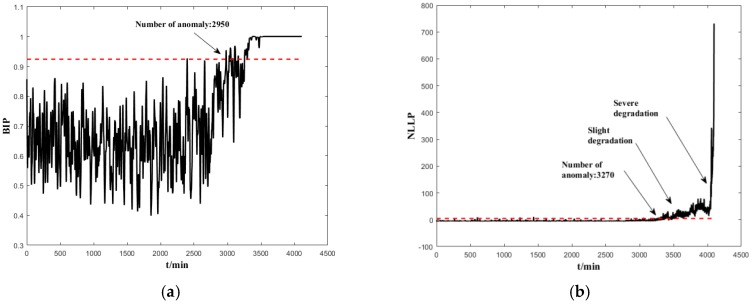
Degradation processes of B1 (**a**) BIP and (**b**) negative log-likelihood probability (NLLP).

To highlight the efficacy of the indicators used in the proposed method, the indicators of the Bayesian-inference-based failure probability (BIP) [28] and negative log-likelihood probability (NLLP) [29] proposed in the literature are used to assess B1. It can be seen from Figure 9 that both indicators are effective in detecting the degradation. However, instead of changing continuously, the BIP value hovers around 1 after detecting the anomaly. Therefore, the BIP is only suitable for anomaly detection, but not for assessment of performance degradation. The following are the comparisons between BID and NLPP: (1) both the indicators are suitable for an accurate assessment of degradation. Both of them revealed the same development trends during the entire life of the bearings and reflected the entire process from normal to initial degradation, severe degradation and the final failure; (2) the BID indicator can detect the degradation earlier than the NLLP indicator, and the stage of degradation is more clear than NLLP.

### 5.2. Comparison with SVDD Assessment Method

**Figure 10:**
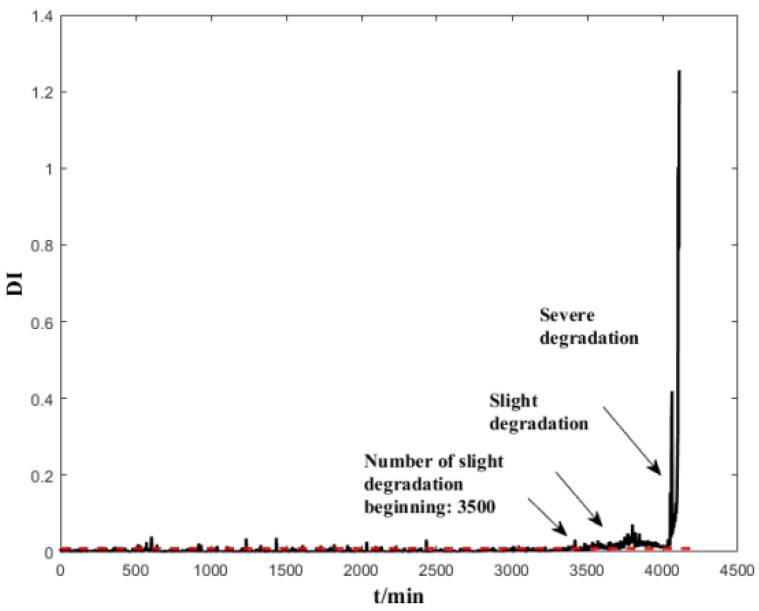
Performance assessment of the whole life time of B1 by SVDD.

The support vector data description (SVDD) is used to assess the performance degradation [30]; the most commonly used Gaussian kernel is chosen as the kernel function, and the Gaussian kernel width parameter is set as σ=5. The SVDD model is trained using normal-state data (the first 200 groups) to calculate the kernel distance between the test data and the hypersphere (see Figure 10). The following are some of the observations: (1) as the distance indicator (DI) often triggers false alarms under normal operating conditions, it is not a reliable indicator; (2) the GMM-based BID indicator can detect the occurrence of bearing performance degradation earlier than the SVDD evaluation result and identify the degradation stages more clear than the SVDD evaluation result.

### 5.3. Comparison of Electrostatic Monitoring and Vibration Monitoring

**Figure 11:**
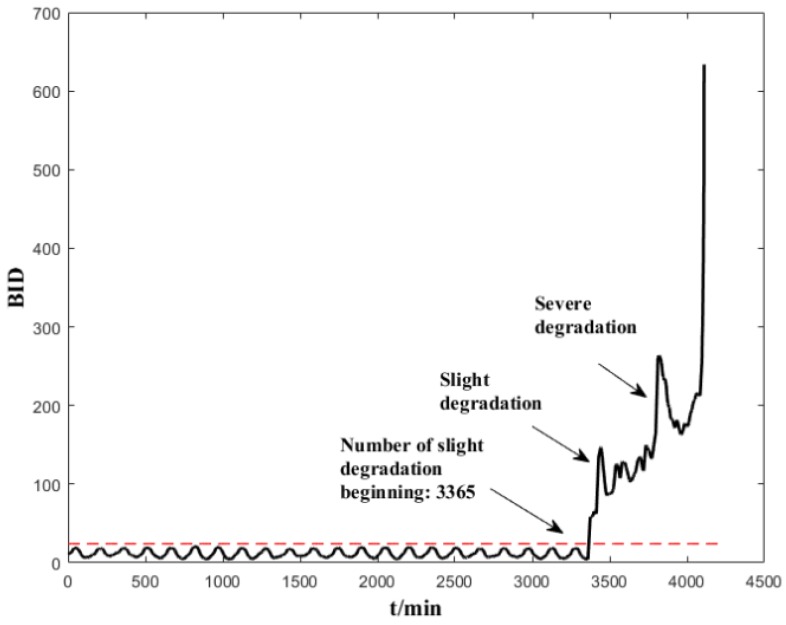
Performance assessment of the whole life time of B1 with vibration monitoring sensors.

In order to compare the two monitoring methods under the same framework, vibration monitoring also adopts the time domain, frequency domain and permutation entropy mentioned in this paper as characteristic features. The method proposed in this paper (SR–GMM–BID) is applied for the performance assessment using the vibration monitoring. As shown in Figure 11, the method can detect the early performance degradation, development of the performance degradation and total failure of the bearing effectively. The difference is that the electrostatic monitoring can detect the anomaly earlier than the vibration monitoring (about 400 min earlier in the case of B1).

Of course, the electrostatic monitoring is not universally applicable. As the electrostatic sensor needs to be fitted closer to the roller bearing, conducting an assessment on an enclosed machine means a hole needs to be drilled on the casing of the machine. Also, the weak electrostatic induction signal can be easily corrupted by the electromagnetic interference in the working environment, triggering false alarms. Therefore, the electrostatic monitoring can be a useful supplement to the conventional monitoring methods such as vibration monitoring and temperature monitoring.

## 6. Conclusions

This paper proposes a new method to assess the performance degradation of bearings based on the fusion of multiple feature parameters in electrostatic monitoring. The experiment results revealed the following:

(1) Compared to the feature extraction methods based on PCA and LPP, the spectral regression has showed better performance in identifying different stages of degradation and requires less computation time;
(2) The permutation entropy serves to extract and amplify small changes in the time sequence, which constitutes a useful complement to the conventional time domain and frequency domain parameters in electrostatic monitoring;
(3) Compared to the NLLP BIP indicators and SVDD evaluation methods, the method (SR–GMM–BID) proposed in this paper can detect the occurrence of performance degradation much earlier;
(4) With the application of the methods proposed in this paper, the electrostatic monitoring can accurately detect early degradation compared to the vibration monitoring, providing more time for making maintenance decisions.

## Figures and Tables

**Table 1:** The traditional feature parameters.

Time-Domain Feature Parameters		Frequency-Domain Feature Parameters	
Feature	Equation	Feature	Equation
Root mean square	$x_{rms}=\sqrt{\frac{\sum_{n=1}^{N}{\left(x(n)\right)}^{2}}{N}}$	Frequency centre	$x_{fc}=\frac{\sum_{k=1}^{K}f_{k}s(k)}{\sum_{k=1}^{K}s(k)}$
Standard deviation	$x_{std}=\sqrt{\frac{\sum_{n=1}^{N}{\left(x(n)-x_{m}\right)}^{2}}{N-1}}$	Root mean square frequency	$x_{rmsf}=\sqrt{\frac{\sum_{k=1}^{K}f_{k}^{2}s(k)}{\sum_{k=1}^{K}s(k)}}$
Peak-Peak	$x_{p-p}=\max(x_{n})-\min(x_{n})$	Standard deviation frequency	$x_{stdf}=\sqrt{\frac{\sum_{k=1}^{K}{\left(f_{k}-x_{fc}\right)}^{2}s(k)}{\sum_{k=1}^{K}s(k)}}$
Skewness	$x_{ske}=\frac{\sum_{n=1}^{N}{\left(x(n)-x_{m}\right)}^{3}}{(N-1)x_{std}^{3}}$	Spectrum peak ratio inner	$SPRI=\frac{k\sum_{i=1}^{H}p_{I}(h)}{\sum_{k=1}^{K}s(k)}$
Kurtosis	$x_{kur}=\frac{\sum_{n=1}^{N}{\left(x(n)-x_{m}\right)}^{4}}{(N-1)x_{std}^{4}}$	Spectrum peak ratio outer	$SPRO=\frac{k\sum_{i=1}^{H}p_{o}(h)}{\sum_{k=1}^{K}s(k)}$
Crest factor	$CF=\frac{x_{p}}{x_{rms}}$	where *s*(*k*) is a spectrum for *k* = 1, 2, …, *K*, *K* is the number of spectrum lines; $f_{k}$ is the frequency value of the *K*th spectrum line; $p_{o}(h)$, $p_{I}(h)$ and $p_{R}(h)$ are, respectively, the peak values of the *h*th (*h* = 1, 2, …, *H*, *H* is the number of harmonics) harmonics of the characteristic frequencies for bearing outer race ($f_{o}$), inner race ($f_{I}$), which can be calculated according to the following equations: $f_{O}=\frac{f_{r}}{2}N_{R}(1-\frac{d}{D}\cos\alpha )$, $f_{I}=\frac{f_{r}}{2}N_{R}(1+\frac{d}{D}\cos\alpha )$. $f_{r}$ is the shaft rotational frequency; $N_{R}$ is the roller number; α is the contact angle; *d* and *D* are the roller and pitch diameters, respectively.	
Impulse factor	$IF=\frac{x_{p}}{\left(\frac{1}{N}\sum_{n=1}^{N}\left|x(n)\right|\right)}$		
Clearance factor	$CLF=\frac{x_{p}}{{\left(\frac{1}{N}\sum_{n=1}^{N}\sqrt{\left|x(n)\right|}\right)}^{2}}$		
Shape factor	$SF=\frac{x_{rms}}{\left(\frac{1}{N}\sum_{n=1}^{N}\left|x(n)\right|\right)}$		
where *x*(*n*) is a signal series for *n* = 1, 2, …, *N*, *N* is the number of data points.			

**Table 2:** Accuracy rate and computation time of classification by K-means for original features, PCA, LPP and SR.

Algorithm	Accuracy Rate (%)	Time (s)
PCA	84.7%	3.059
LPP	96%	3.276
SR	100%	1.814
